# Hydrocarbon Cycling in the Tokamachi Mud Volcano (Japan): Insights from Isotopologue and Metataxonomic Analyses

**DOI:** 10.3390/microorganisms10071417

**Published:** 2022-07-14

**Authors:** Alexis Gilbert, Mayuko Nakagawa, Koudai Taguchi, Naizhong Zhang, Akifumi Nishida, Naohiro Yoshida

**Affiliations:** 1 Department of Earth and Planetary Sciences, Tokyo Institute of Technology, Tokyo 152-8550, Japan; nakagawa.m.ae@m.titech.ac.jp (M.N.); taguchi.k.ab@m.titech.ac.jp (K.T.); zhang.n.aa@m.titech.ac.jp (N.Z.); 2 Earth-Life Science Institute, Tokyo Institute of Technology, Tokyo 152-8550, Japan; naohiroyoshida@elsi.jp; 3 Department of Molecular Microbiology, Tokyo University of Agriculture, Tokyo 156-8302, Japan; an207919@nodai.ac.jp

**Keywords:** stable isotopes, hydrocarbons, microorganism, position-specific isotope analysis, metagenomics

## Abstract

Understanding hydrocarbon cycling in the subsurface is important in various disciplines including climate science, energy resources and astrobiology. Mud volcanoes provide insights into biogeochemical processes occurring in the subsurface. They are usually associated with natural gas reservoirs consisting mainly of methane and other hydrocarbons as well as CO_2_. Stable isotopes have been used to decipher the sources and sinks of hydrocarbons in the subsurface, although the interpretation can be ambiguous due to the numerous processes involved. Here we report new data for hydrocarbon isotope analysis, including position-specific isotope composition of propane, for samples from the Tokamachi mud volcano area, Japan. The data suggest that C_2+_ hydrocarbons are being biodegraded, with indirect production of methane (“secondary methanogenesis”). Data from chemical and isotopic composition are discussed with regard to 16S rRNA analysis, which exhibits the presence of hydrogenotrophic and acetoclastic methoanogens. Overall, the combination of isotopologue analysis with 16S rRNA gene data allows refining of our understanding of hydrocarbon cycling in subsurface environments.

## 1. Introduction

Mud volcanoes are surficial cone-shaped landforms formed by the expulsion of fluids from deep-seated subsurface layers [1]. The fluids expulsed include gas and water as well as fine-grained minerals which, mixed with water, yield mud, hence their name. Because the material discharged comes from depths down to a few kms, mud volcanoes can provide invaluable insights into biogeochemical processes occurring in the subsurface without the need for expensive drilling [2,3,4]. Furthermore, similar structures have been observed on Mars and are considered potential candidates for future exploration missions aiming at traces of life from the subsurface of Mars [5,6,7,8]. Finally, the gases discharged from mud volcanoes, whereas variable from one site to another, are mainly dominated by methane, making them important contributors to the natural hydrocarbon budget in the atmosphere [1,9].

Chemical and stable isotope composition of the hydrocarbons emitted from mud volcanoes can inform on the processes occurring in the subsurface. In particular, the δ^2^H and δ^13^C values of methane, in combination with the relative CH_4_ content of the gas (usually expressed as the ratio C_1_/(C_2_ + C_3_)) have been used to distinguish microbial methane from thermogenic methane (for a review, see [10]). Yet, the interpretation of the stable isotope and chemical composition is made complicated by several factors: (i) the stable isotope and chemical signatures can vary considerably even within a single type of process (ii) most natural gases occur as mixtures between two or more end-members and (iii) post-genetic or “secondary” processes such as migration, diffusion or biodegradation can significantly alter the primary signature of the gases.

The past few years have witnessed a boom in hydrocarbon isotopic analysis with the advent of isotopologue analysis. Isotopologues are molecules that differ in the number and/or position of isotope substitution [11,12]. These include multiply substituted isotope species such as **^13^C**H_3_**D** and CH_2_**D_2_** for methane [13,14,15,16], **^13^C**H_3_-**^13^C**H_3_ for ethane [17,18], as well as position-specific isotopologues such as **^13^C**H_3_-CH_2_-CH_3_ and CH_3_-**^13^C**H_2_CH_3_ of propane [19,20,21,22]. The latter approach has proven particularly useful for the detection of hydrocarbon anaerobic oxidation in natural gas basins [23]. Through culture experiments using sulfate-reducing bacteria isolated from the Guaymas Basin [24], Gilbert et al. [23] showed that bacteria tend to assimilate the central ^13^C-isotopologue of propane at a lower rate compared with the terminal ^13^C-isotopologue and the ^12^C-isotopologue. The relative ^13^C-enrichment depends on the extent of anaerobic oxidation of propane and has been used to detect and quantify biodegradation of propane in natural gas reservoirs [12,23].

This paper proposes a reconnaissance study taking advantage of these new developments to shed light on hydrocarbon cycling in the subsurface of the Tokamachi mud volcanoes located in the Niigata prefecture in Northern Japan [25,26,27,28]. The geochemistry of the Tokamachi mud volcanoes has been studied previously [26,27,28] and gas sample analysis suggested biological activity, notably anaerobic oxidation of hydrocarbons [25]. These geological structures are thus an interesting setting to study subsurface biological cycling. Here, we use conventional chemical and isotopic analyses as well as position-specific ^13^C isotope analysis of propane to investigate hydrocarbon oxidation in Tokamachi mud volcanoes. We first confirm the occurrence of anaerobic oxidation of propane using position-specific ^13^C isotope composition. Then we relate the latter to the isotope composition of other hydrocarbons, namely, methane and ethane, to constrain further their sources and sinks. Finally, we couple our approach with metataxonomic approaches, i.e., 16S rRNA gene analysis, to complement the interpretation obtained through isotopologue analysis.

## 2. Materials and Methods

### 2.1. Study Site and Sampling Points

**Figure 1:**
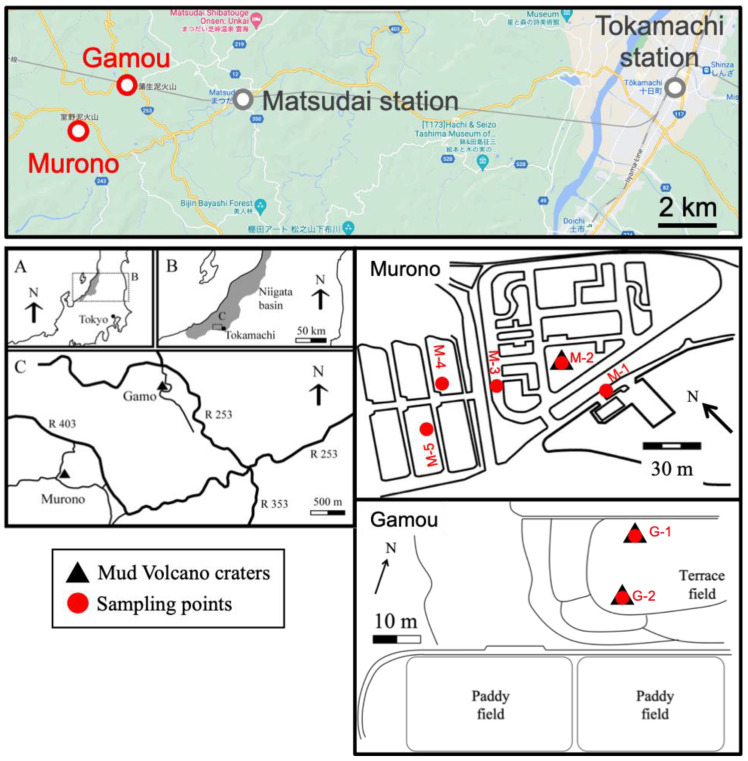
Maps of the Tokamachi area including 2 sampling sites (Murono and Gamou). Samples were taken from mud volcano craters (black triangles) as well as seeping points. Sampling points are indicated as red circles. The upper map is a screenshot from Google Maps accessed on 8th July 2022.

**Figure 2:**
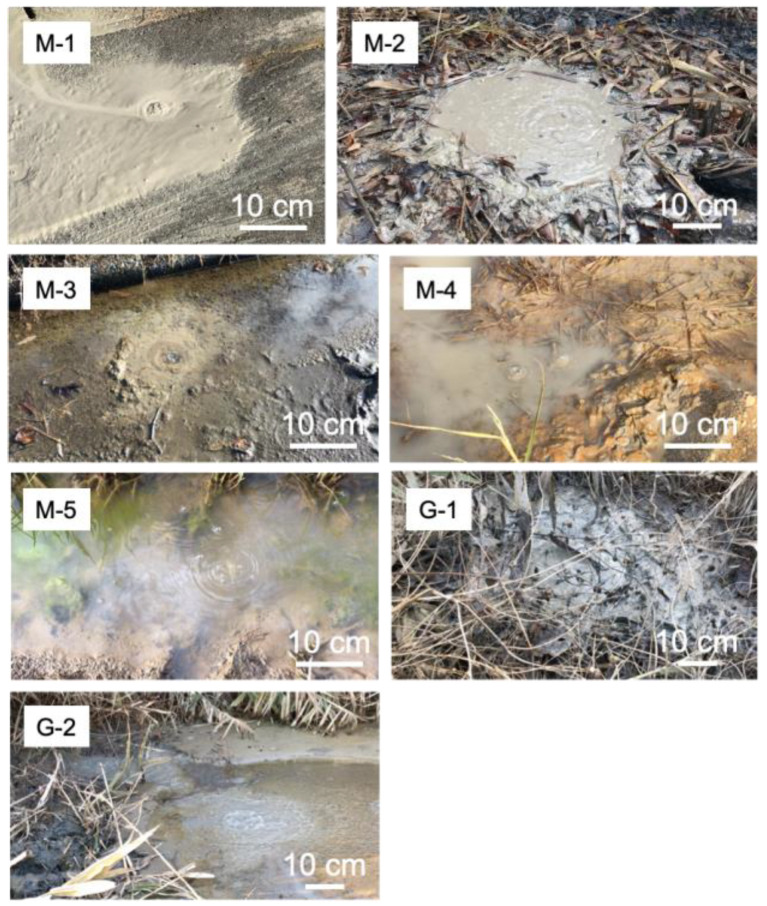
Pictures taken at the 7 sampling points indicated in Figure 1. The sampling points (M-1, M-2, M-3, M-4, M-5, G-1 and G-2) are those described in Figure 1.

The Tokamachi area is located in the Niigata prefecture, Japan (Figure 1; [25]). The area consists of two main locations, Murono and Gamou, located ca. 2 kms away from each other. The coordinates for the Murono and Gamou mud volcanoes are [37.121169, 138.558106] and [37.133696, 138.577632], respectively. The area has three mud volcano craters and several spots where gas seeps naturally. In total, seven samples were taken: three from the mud volcanoes (two from Gamou, one from Murono) and four from seeping points on Murono (Figure 2). Water temperatures varied between 10.6 °C and 15.7 °C. The temperature at each sampling point is reported in Table S1. The pH was not measured in the present investigation but previous studies reported a slightly alkaline pH, around 7.5 [26]. Methane fluxes from the area have been measured previously [25] and are up to 10^4^ g.m^−2^.day^−1^ and 446 g.m^−2^.day^−1^ for mud volcanoes and microseepages, respectively.

Gas samples were taken following the water displacement method [29]. Briefly, the gas was collected using a glass funnel (10 cm i.d.) where the end is connected to a plastic tube. The other end of the plastic tube is placed in a water tank. The bubbles are directed into a vial filled with water and placed upside down into the water tank. As the bubbles fill the vial with gas, water is displaced outside of the vial. Once enough gas has been collected (typically > 80% of the vial), the vial is closed with a rubber septum and crimped with an aluminum cap. The mud samples were sterilely collected in plastic centrifuge tubes and kept in an ice box until they were transferred to the lab, then stored frozen (−30 °C) until molecular analysis.

### 2.2. DNA Extraction and Bioinformatic Analysis

Genomic DNA in mud volcanoes was isolated using the DNeasy PowerSoil DNA Isolation kit (Qiagen, Hilden, Germany). DNA purity was assessed using a Nanodrop spectrophotometer (NanoDrop Technologies Inc., Wilmington, DE, USA). DNA samples were sent to the Integrated Microbiome Resource (IMR), Centre for Comparative Genomics and Evolutionary Bioinformatics, Dalhousie University (Canada) for sequencing. Bacterial diversity was characterized via PCR amplification of the 16S rRNA gene (V4-V5 region) using barcoded primers 515F and 926R [30]. Sequencing of the amplicons was conducted at IMR using the paired-end (2 × 300 bp) Illumina MiSeq system (Illumina, San Diego, CA, USA). Sequencing data were deposited at the National Center for Biotechnology Information (Bethesda, Rockville, MD, USA) (Accession number: PRJNA854688).

QIIME2 docker software (qiime2-2019.10) was used to analyze the 16S rRNA gene sequences [31]. Mud volcano 300 nucleotide (nt) paired-end reads were trimmed using the *qiime dada2 denoise-paired* command (20–296 nt for forward and 20–250 nt for reverse). The reads were taxonomically classified with 99% amplicon sequence variant (ASV) data using SILVA 132 [32] and the *qiime feature-classifier extract-reads* command (GTGYCAGCMGCCGCGGTAA for forward and CCGYCAATTYMTTTRAGTTT for reverse). The relative abundance at the genus level from the ASV taxonomy data is shown in Table S2.

### 2.3. Bulk Isotope Analysis of Hydrocarbons and CO_2_

The ^13^C composition of hydrocarbons (methane, ethane, propane, *i*-butane, *n*-butane) and CO_2_ was determined using a gas chromatograph coupled with an isotope ratio mass spectrometer (DeltaplusXP, Thermo Fisher Scientific, Waltham, MA, USA) via a combustion furnace and a conflow interface (GC Combustion III, Thermo Fisher Scientific) [23]. High-purity helium was used as the carrier gas. The conditions of the GC oven were as follows: injector temperature 250 °C; split mode (split ratio = 80–100 for methane; split ratio = 10 for other molecules); flow rate 1.5 mL/min; oven temperature program 50 °C (maintained 5 min) raised to 200 °C (maintained 10 min) at a rate of 10 °C/min. The column used was an HP-PLOT-Q (30 m × 0.32 mm i.d., 10 µm film thickness; Varian, CA, USA). The effluent was then introduced into a combustion furnace (ceramic tube packed with CuO, NiO, and Pt wires; operating at 960 °C) before being analyzed by the IRMS. Isotopic standardization was made by CO_2_ injections calibrated against the NIST natural gas standard NGS-2 [33]. Standard deviations from 3−5 measurements were generally lower than 0.5‰.

The bulk δD isotope values of CH_4_ were analyzed using a Thermo 253 Ultra Isotope Ratio Mass Spectrometer (Ultra-IRMS; Thermo Fisher Scientific Inc.) at Tokyo Institute of Technology. Sample preparation and analytical protocols followed the exact same procedures as described in [34], which is briefly listed here. Approximately 4–6 mL mud volcano sample was purified using a gas chromatograph (GC-4000, GL-Science) with He as the carrier gas. Pure CH_4_ gas was separated and collected in a 2 mL silica-gel-filled stainless finger using a liquid nitrogen trap, which was subsequently introduced into the sampling bellow of the Ultra. At the HR+ (high resolution +) mode, the [^12^CH_3_D+] peak can be fully separated from other adduct peaks and its intensity was registered using an H4 CDD collector equipped on the Ultra-IRMS. The internal precision of δD (1σ-SE) is typically better than 0.15‰.

### 2.4. Position-Specific ^13^C Isotope Analysis of Propane

For position-specific ^13^C isotope analysis of propane, samples were introduced using a gas-tight syringe into an on-line pyrolysis system coupled with GC-C-IRMS, as previously described in Gilbert et al. [19,23]. High-purity helium was used as the carrier gas. A first GC column (HP-PLOT-Q, 30 m × 0.32 mm i.d., 10 µm film thickness; Varian, CA, USA) was connected to a high-temperature conversion furnace (deactivated fused-silica capillary column 0.32 mm i.d. inserted in a ceramic tube of 25 cm × 0.5 mm i.d., operating at different temperatures) to pyrolyze propane at a temperature of 825 °C. The pyrolytic fragments (CH_4_, C_2_H_4_ and C_2_H_6_) were separated on a second GC capillary column (HP-PLOT-Q, 30 m × 0.32 mm i.d., 10 µm film thickness; Varian, CA, USA), and introduced into a combustion furnace (ceramic tube packed with CuO, NiO and Pt wires; operating at 960 °C) before being analyzed by the IRMS (Delta XP, Thermo Fisher Scientific Inc., Waltham, MA, USA). The conditions of the first GC oven were as follows: injector temperature 250 °C; split mode (split ratio = 1); flow rate 3.5 mL/min; oven temperature program 50 °C (10 min) raised to 200 °C (9 min) at a rate of 10 °C/min. The second GC oven was kept at 50 °C throughout the analysis. Once the C_1_ and C_2_ fragments from pyrolysis of hydrocarbons were eluted from the second column the temperature of the second GC oven was raised to 200 °C at 20 °C/min in order to elute unreacted hydrocarbons. The connections between the GC columns and the pyrolysis and combustion furnaces were made using a deactivated fused-silica capillary column (0.32 mm i.d.). Isotopic standardization was made by CO_2_ injections calibrated against the NIST natural gas standard NGS-2 [33]. The accuracy of the system was checked by regularly injecting working standards. The relative enrichment in the central position (∆^13^C_Central_, in ‰) is defined as the difference in isotopic composition between the central and terminal positions: ∆^13^C_Central_ = δ^13^C_Central_ − δ^13^C_Terminal_. Three fragments are used for its calculation: CH_4_, C_2_H_4_ and C_2_H_6_. CH_4_ and C_2_H_6_ arise from the terminal position only, whereas C_2_H_4_ arises from an equal contribution of terminal and central positions [19]. The isotope composition of the terminal position of propane can be calculated as follows:

δ^13^C_Terminal_ = δ^13^C_CH4, original_ (1)

with

δ^13^C_CH4,original_ = (δ^13^C_CH4_ ∗ A_CH4_ + δ^13^C_C2H6_ ∗ A_C2H6_)/(A_CH4_ + A_C2H6_) (2)

where A is the area of the fragment peaks. Then, the isotope composition of the central position can be calculated:

δ^13^C_Central_ = 2 (δ^13^C_C2H4_) − δ^13^C_Terminal_ (3)

The relative ^13^C-enrichment on the central position then becomes:

∆^13^C_Central_ = δ^13^C_Central_ − δ^13^C_Terminal_ (4)

Standard deviations from the mean ∆^13^C_Central_ value from 3–5 measurements were lower than 1.5‰.

## 3. Results and Discussion

### 3.1. Bulk and Intramolecular Isotope and Chemical Composition

The natural gas emitted from the Tokamachi area consists mainly of methane (93.19% to 96.66%), followed by CO_2_ (2.07% to 5.95%) with small proportion of C_2+_ hydrocarbons (0.05% to 0.76% for ethane and 0.002% to 0.32% for propane) (Table 1).

**Figure 3:**
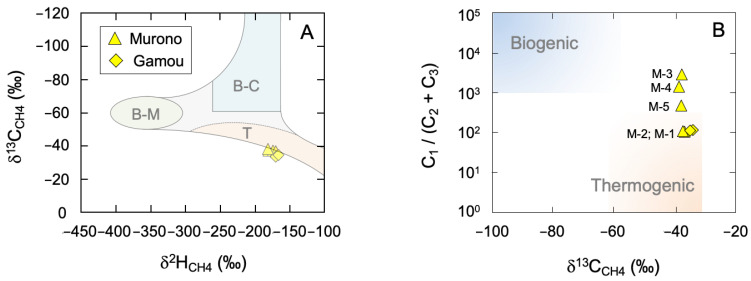
Conventional diagrams for the diagnosis of methane sources: (**A**) δ^2^H vs. δ^13^C (“Schoell diagram”) (**B**) δ^13^C vs. C_1_ / (C_2_ + C_3_) (“Bernard plot”). “T”: Thermogenic; “B-M”: Biogenic (Methyl-type fermentation); “B-C”: Biogenic (CO_2_ reduction). Error bars are within the symbols. Sample numbers for Murono are indicated on (**B**).

Methane δ^2^H and δ^13^C values (Figure 3A) span a narrow range and indicate a thermogenic origin, consistent with previous measurements [25] and with the fact that the Tokamachi area is located over the Niigata natural gas basin. A Bernard diagram (Figure 3B) shows variable C_1_/(C_2_ + C_3_) ratios, suggesting that methane is thermogenic in origin but that the gas has been altered either by a process removing C_2+_ hydrocarbons (e.g., preferential biodegradation) or by a process enriching methane specifically (e.g., preferential migration or methanogenesis), or both.

### 3.2. Position-Specific ^13^C Composition of Propane

**Figure 4:**
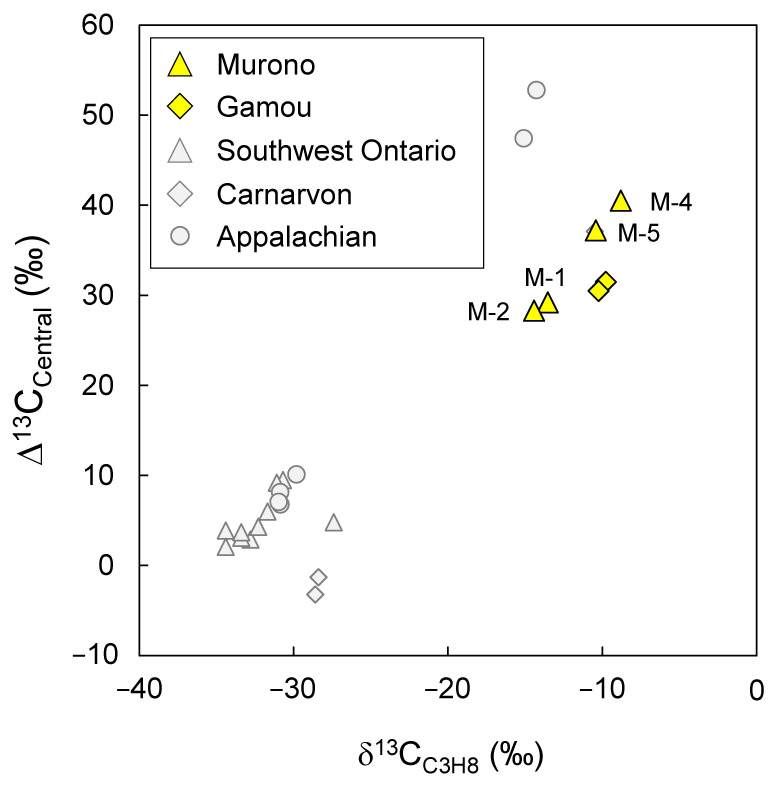
Relative ^13^C-enrichment on the central position (∆^13^C_Central_) versus bulk isotope composition (δ^13^C_C3H8_) of propane from Tokamachi mud volcano area (Murono and Gamou). Error bars are within the symbol. Gray symbols are samples from previous studies including samples from the Appalachian Basin (USA), Southwest Ontario (Canada) and Carnarvon (Australia) [19,23]. Sample numbers for Murono are indicated.

The δ^13^C values of propane range from −14.4‰ to −8.8‰ (Figure 4) and are in the same range as those previously reported values by Etiope et al. [25]. These values are higher than known thermogenic samples and suggest post-genetic alteration of propane. The high δ^13^C_C3H8_ values correspond to high ^13^C enrichment on the central position of propane ∆^13^C_Central_ (Table 2; Figure 4). Indeed, the ^13^C-enrichment of propane is virtually located solely on the central position with δ^13^C_Central_ values up to +18.2‰ (Table 2), whereas the terminal position is barely affected. This enrichment is consistent with the anaerobic oxidation of propane. Bacteria activate propane on the central position, hence the central ^13^C-isotopologue tends to react slower compared with the terminal ^13^C-isotpologue or the fully ^12^C-isotopologue. As a result, the isotope composition of the central position increases with the degree of biodegradation. Gilbert et al. [23] measured isotope fractionation associated with propane biodegradation by bacteria BuS5 isolated from the Guaymas Basin [24]. The isotope fractionation factors are 33‰ and 3‰ for the central and terminal position of propane, respectively. A strong ^13^C-enrichment on the central position of propane has thus been suggested to be an indicator of anaerobic oxidation [23].

Based on bacterial isotope fractionation factors measured on pure cultures, and assuming the starting propane is a thermogenic sample with no relative ^13^C-enrichment (∆^13^C_Central_ = 0), the extent of biodegradation in the Tokamachi area varies from 55% to 71%. Whereas the Murono samples show a linear trend between ∆^13^C_Central_ and δ^13^C_C3H8_, the Gamou samples appear slightly shifted. A linear trend, as observed for the Murono samples, implies that the samples shared the same origin and the same biodegradation mechanism (same position-specific isotope fractionation). On the other hand, the fact that Murono and Gamou samples are not aligned in Figure 4 suggests either that their original source is different and/or that the isotope fractionation associated with their biodegradation is different. The former is likely and has been suggested to explain the difference between samples from the Carnarvon Basin (Australia) and the Appalachian Basin (USA) (Figure 4 and see ref. [23]). Previous studies have also demonstrated that differences in the bulk and position-specific ^13^C isotope composition of propane can be influenced by the source, the rate and temperature of gas formation as well as secondary processes such as migration or diffusion [21,35,36]. A different original source of hydrocarbons is supported by the higher ^13^C/^12^C ratios for methane and ethane in Gamou compared with those in Murono (Table 2).

In addition, it is possible that the fractionation factors associated with hydrocarbon biodegradation differs between the two environments, since several factors can influence isotope fractionation by microorganisms. These include temperature, mechanism of assimilation and the concentration of nutrient. The only enriched cultures of microorganisms able to oxidize propane in anaerobic conditions are the sulfate-reducing bacteria BuS5 [24] and the archaea from the proposed genus ‘*Candidatus* Syntrophoarchaeum’ operating in syntrophy with sulfate-reducing bacteria [37]. Whereas isotope fractionation factors have been determined for the bacteria BuS5 [23], they are currently unknown for the archaea. Given that the mechanisms of propane activation are different for bacteria and archaea (fumarate addition to the central C-atom for the former [24], activation by co-enzyme M to the terminal C-atom for the latter [37]), it is likely that the fractionation factors are different. In addition, isotope fractionation factors for BuS5 bacteria have only been measured in a single set of conditions, and it is possible that the conditions (propane concentration, temperature, …) influence the isotope fractionation factors. These elements may contribute to a discrepancy between pure culture experiments and field data, as well as a discrepancy between two reservoirs with different microbial compositions or environmental conditions. The temperatures measured at Gamou and Murono surface waters are not statistically significant (Table S1), making it difficult to invoke temperature as a determining factor to explain differences in isotope fractionation factors associated with microbial oxidation. On the other hand, microbial composition might play a role, but as long as the microorganisms degrading propane are not identified (e.g., using culture experiments), it is difficult to draw conclusions about their implications in the discrepancies observed between Murono and Gamou. Clearly therefore, isotope fractionation factors for the archaea, as well as the influence of environmental conditions on isotope fractionation must be studied in order to correctly interpret the isotope composition of propane from natural samples. This will be the object of further studies.

The trend observed for Murono samples in Figure 4 represents either (i) samples ejected from different strata/reservoirs with various degrees of biodegradation (and thus different degrees of isotopic enrichment) or (ii) different degrees of mixing between two end-members: a thermogenic non-altered, deep-seated reservoir and a shallower reservoir where propane is highly biodegraded. In any case, the Murono area represents a sequence with various degrees of propane biodegradation, making it a unique opportunity to study propane biodegradation in connection to other hydrocarbons.

### 3.3. Is Ethane Also Biodegraded?

**Figure 5:**
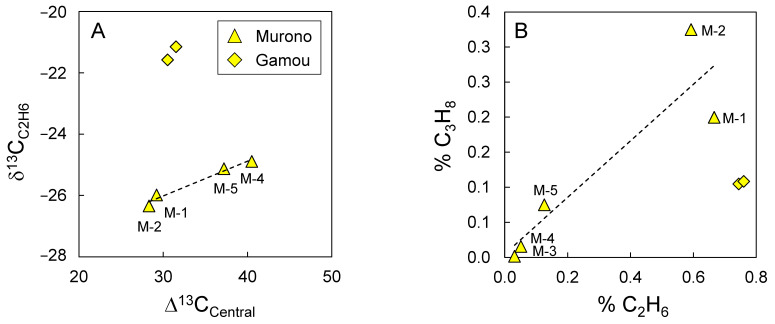
Relationship between ethane and propane: (**A**) isotope composition and (**B**) relative concentration %. Dotted lines indicate best fit for linear regression for Murono samples only. Sample numbers for Murono are indicated.

Propane is often considered the most biodegraded natural gas hydrocarbon (e.g., [38]), followed by *n*-butane, ethane and *i*-butane. *n*-Butane is below our detection limit in the Murono samples. However, ethane is detected and shows δ^13^C values that correlate with those of propane (Figure 5A). This suggests that the biodegradation process also affects ethane. The relative concentration of ethane also varies with that of propane (Figure 5B), further supporting the co-biodegradation of propane and ethane. The slope of Figure 5B is 0.4, suggesting that the rate of ethane biodegradation is ca. 40% that of propane. Despite this relatively high rate of biodegradation, ethane isotope composition varies only slightly compared to that of propane, which could partly explain why biodegradation of ethane is harder to detect. The low isotope fractionation factor for ethane biodegradation could be due to the mechanism of activation. The only microorganism thus far known to degrade ethane activates ethane on one of the CH_3_ positions with co-enzyme M [39], whereas the bacteria, BuS5, activate CH_2_ positions using alkyl succinate synthase. Importantly, the isolated archaea that degrades propane activates propane with co-enzyme M. Hence, it is possible that the isotope fractionation factor differs between bacteria using succinate synthase and archaea using co-enzyme M. In such a scenario, propane would have apparent isotope fractionation between that of bacteria and that of archaea where ethane would reflect that of archaea only, since no bacteria is known to oxidize ethane. If the isotope fractionation for archaea is small, the apparent isotope fractionation would be lower for ethane than for propane. This study would thus benefit from additional efforts to determine the isotope fractionation factors associated with archaeal anaerobic hydrocarbon oxidation.

### 3.4. Evidence for Secondary Microbial Methane

**Figure 6:**
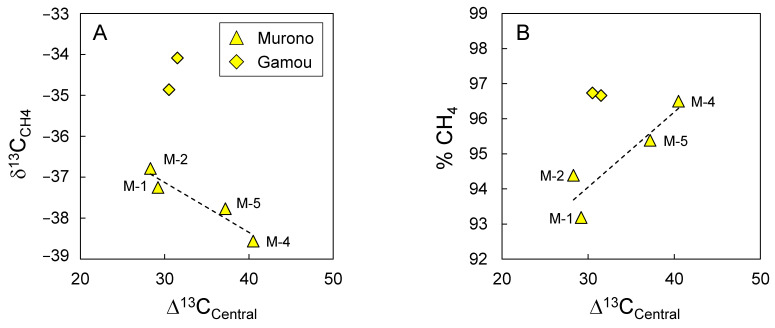
Relationship between the ∆^13^C_Central_ of propane and (**A**) the isotope composition of methane and (**B**) the relative concentration of methane (% CH_4_). Dotted lines indicate best fit for linear regression for Murono samples only. Sample numbers for Murono are indicated.

Whereas the extent of biodegradation (represented here as the ∆^13^C_Central_ value of propane) is positively correlated with δ^13^C values of ethane, an inverse correlation is observed for methane (Figure 6A). In addition, the relative concentration of methane increases with the extent of biodegradation (Figure 6B). These two facts taken together suggest that the biodegradation of propane is associated with methane formation. This is consistent with the so-called secondary microbial methane generation during which methane is produced from products of hydrocarbon anaerobic oxidation, either directly or through acetate or CO_2_ (for a comprehensive review, see [40]).

Secondary microbial methane is generally associated with high δ^13^C values for CO_2_. The ^13^C-enrichment has been proposed to arise from a normal (^12^C-preferred) isotope effect during CO_2_ reassimilation by methanogens, resulting in remaining ^13^C-enriched CO_2_. In both Gamou and Murono these values are high, ranging from + 19.1‰ to + 34.5‰ (Table 2), which is consistent with the generation of secondary microbial methane from CO_2_ [40].

### 3.5. Microbial Community Composition and Members Involved in Hydrocarbon Degradation

**Figure 7:**
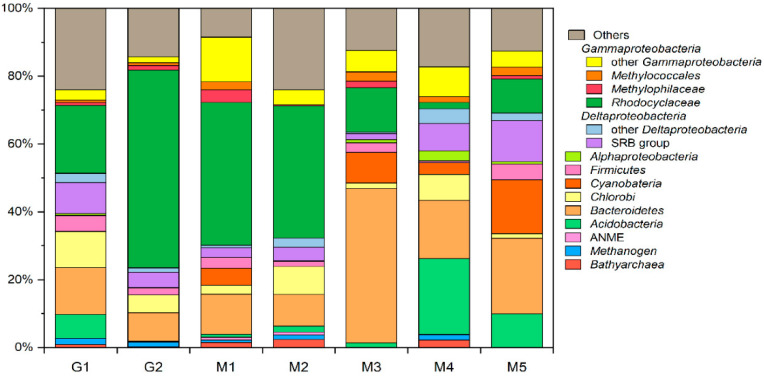
Relative abundance of community members in Gamou (‘G’) and Murono (‘M’) samples.

Results of 16S rRNA gene amplicon sequencing indicate that the microbial communities were different between Gamou and Murono (Figure 7, Table S2). The microbial community contained 0.1–10% archaea. The archaeal compositions were represented by members of Bathyarchaeota, Euryarchaeota (Methanomicrobia and Themoplasmata) and Candidatus Woesearchaeia. Methanogens were dominant in these microbial compositions. The most abundant phyla were Bathyarchaeota and other archaeal phyla including methanogens of the orders *Methanomicrobiales*, *Methanosarcinales*, *Methanomassiliicoccales*, as well as Candidatus Methanomethylicales and the genus *Candidatus* Methanofastidiosa. The most abundant bacterial phyla were Proteobacteria (>30% of all reads) and Bacteroidetes (>8%). The Proteobacteria in samples were mainly Gammaproteobacteria, with the dominant family being *Rhodocyclaceae*, Deltaproteobacteria and Alphaproteobacteria. A few percent of the microbials in Gammaproteobacteria belong to the family *Methylophilaceae* and *Burkholderiaceae* of the order *Methylococcales*. The Deltaproteobacteria is mostly composed of sulfate-reducing bacteria belonging to the order *Desulfuromonadales*, *Desulfobacteriales*, *Desulfovibrionales*, *Desulfarculates* and *Syntrophobacteriales*. The order *Rhodobacteriales* and *Rhizobiales* dominated the Alphaproteobacteria. Other representatives of this class were related to the order *Sphingomonadales*, *Acetobacterales*.

From the isotope and chemical composition of gases (see previous paragraphs), propane and possibly ethane are oxidized. The only isolated culture that is an anaerobic propane oxidizer is the sulfate-reducing bacterium ‘*Desulfosarcina aeriophaga’*, which was isolated from the Guaymas Basin [24,41]. However, the archaea ‘*Candidatus* Syntrophoarchaeum’ from the enriched cultures of the Guaymas Basin was recently shown to oxidize butane and propane anaerobically through syntrophy with a sulfate-reducing bacteria [37]. In any case, microorganisms conducting anaerobic oxidation of propane and butane have been shown to use sulfate as an electron acceptor [41], and to date no microorganisms oxidizing gaseous C_2+_ hydrocarbons with other electron acceptors have been identified. BuS5 was not detected in either the Gamou or Murono samples, although sulfate reducers are clearly present.

There were some potential alternative candidates for hydrocarbon anaerobic oxidation. For instance, the family *Rhodocyclaceae* have been shown to use alkanes with nitrate as the electron acceptor [42]. Furthermore, the *Geobacter* species, which were the main member in the order *Desulfomonadales*, can oxidize hydrocarbons coupling with iron reduction [43,44]. Both of theses are detected in Tokamachi samples and further studies, including incubation of the mud with different electron acceptors (sulfate, nitrate and iron (III)) will give insights into the actual hydrocarbon oxidizing organisms present in the Tokamachi area. Interestingly, anaerobic methanotrophic (ANME) archaea were detected in only a limited amount (<0.9%) in M1 and M2 and were not detected in other samples. Therefore, the anaerobic oxidation of hydrocarbons seems to be limited to hydrocarbons with a chain length > 2, i.e., starting with ethane. The CO_2_ produced could then be used by methanogenic archaea along with H_2_ to produce methane. Hydrogenotrophic methanogens such as *Methanoregula*, *Methanobacterium* and *Methanolinea* are detected in all of the samples in Tokamachi, suggesting secondary methanogenesis from CO_2_ and H_2_ is plausible.

Whereas the short-chain hydrocarbon degrading bacteria and archaea isolated so far produce CO_2_, some long-chain alkane degraders also directly produce methane [45] or acetate [46] for instance. Whether similar microorganisms can grow on short-chain alkanes remains unknown, but they could be a source of methane either directly through methanogenic alkane degradation or indirectly through the fermentation of acetate by acetoclastic methanogens.

## 4. Conclusions

We have investigated the chemical, isotopic and microbial composition of the fluids ejected from mud volcanoes and gas seepages in the Tokamachi area, Japan. Isotopic data suggest propane and other C_2+_ hydrocarbons are being oxidized by anaerobic organisms. Thus far, microorganisms oxidizing short-chain hydrocarbons anaerobically use sulfate as an electron acceptor, although 16S rRNA gene sequences exhibit the presence of nitrate- and iron-reducing bacteria that could play a role in the process. Secondary methanogenesis seems to occur through the fermentation of acetate or the hydrogenotrophic reduction of CO_2_. The preliminary investigation presented here highlights the need for a combination of approaches to decipher hydrocarbon cycling in the subsurface.

Further studies should include culture experiments with different substrates and electron acceptors as well as the analysis of the ion concentrations in the samples (especially sulfate, nitrate and iron species) to determine their role in hydrocarbon production and consumption. Finally, the study should be pursued including newly developed isotopologue measurements such as the ^13^C−^13^C isotopologue of ethane [17,18,47] and the ^13^CH_3_D and CH_2_D_2_ isotopologues of methane [14,15,34,48]. These new tracers are currently used to understand the sources and sinks of hydrocarbons. Mud volcanoes and gas seepages from the Tokamachi area exhibit different degrees of biodegradation and potential secondary methanogenesis, making it a place of choice to study the effect of hydrocarbon production and biodegradation on hydrocarbon isotopologue signatures. This will be addressed in a subsequent study.

## Figures and Tables

**Table 1:** Chemical composition (in %) of the major constituents of gas samples from Murono (M) and Gamou (G) samples. “bdl” = below detection limit.

Sample	CH_4_	CO_2_	C_2_H_6_	C_3_H_8_	*i*-C_4_H_10_	*n*-C_4_H_10_
G-1	96.66	2.18	0.74	0.10	0.26	0.05
G-2	96.74	2.07	0.76	0.11	0.28	0.05
M-1	93.19	5.95	0.67	0.20	bdl	bdl
M-2	94.39	4.70	0.59	0.32	bdl	bdl
M-3	93.94	6.02	0.03	0.002	bdl	bdl
M-4	96.50	3.43	0.05	0.02	bdl	bdl
M-5	95.38	4.41	0.13	0.07	bdl	bdl

**Table 2:** Bulk and position-specific isotope composition of hydrocarbons and CO_2_ from Murono (M) and Gamou (G) samples. “bdl” = below detection limit.

Sample	δ^13^C_CH4_	δD_CH4_	δ^13^C_CO2_	δ^13^C_C2H6_	δ^13^C_C3H8_	δ^13^C*_i_*_-C4H10_	δ^13^C*_n_*_-C4H10_		C_3_H_8_	
								∆^13^C_Central_	δ^13^C_Central_	δ^13^C_Terminal_
G-1	−34.1	−170	28.5	−21.2	−9.8	−24.3	−14.6	31.5	11.2	−20.3
G-2	−34.9	−167	24.4	−21.6	−10.2	−25.2	−15.3	30.5	10.1	−20.4
M-1	−37.2	−170	34.5	−26.0	−13.5	bdl	bdl	29.2	5.9	−23.3
M-2	−36.8	−170	34.1	−26.3	−14.4	bdl	bdl	28.3	4.4	−23.9
M-3	−37.0	−181	32.7	−23.3	−1.7	bdl	bdl	bdl	-	-
M-4	−38.6	−182	19.1	−24.9	−8.8	bdl	bdl	40.5	18.2	−22.3
M-5	−37.8	−175	33.8	−25.1	−10.4	bdl	bdl	37.2	14.4	−22.8

## Data Availability

Not applicable.

## Supplementary Materials

The following are available online at https://www.mdpi.com/article/10.3390/microorganisms10071417/s1, Table S1: chemical, isotopic and microbial compositions of samples from Tokamachi mud volcano area; Table S2 Relative microbial frequency data for each sample by 16S rRNA gene sequencing.
